# Toxicity across food webs: effects of karanja oil on greenhouse whitefly on tomato and two commercial biocontrol agents

**DOI:** 10.3389/finsc.2026.1711812

**Published:** 2026-02-04

**Authors:** César Cabra-Arias, Juli Carrillo

**Affiliations:** 1 Centre for Sustainable Food Systems, Faculty of Land and Food Systems, The University of British Columbia, Vancouver, BC, Canada; 2 Department of Zoology, Biodiversity Research Centre, The University of British Columbia, Vancouver, BC, Canada; 3 Department of Applied Biology, Vancouver, BC, Canada

**Keywords:** botanical extractions, insect pest ecology, karanjin, parasitic wasps, pest control, predatory beetles

## Abstract

Integrated pest management programs often incorporate insect natural enemies for biological control alongside the application of pesticides. However, pesticides can directly affect the survival and performance of beneficial predaceous insects, decreasing their efficacy. Conversely, pesticides may also increase the susceptibility of insect pests to biological control, promoting the predatory performance of their natural enemies. Pest management can be improved by understanding the ecological interaction among plants, pests, and predators, as well as the influence of pesticide application on higher trophic levels. We conducted bioassays on tomato plants *(Solanum lycopersicum* L.) to evaluate the effects of karanja oil, a presumed insecticidal botanical extract obtained from the seeds of the tree *Pongamia pinnata L*, on the greenhouse whitefly *Trialeurodes vaporariorum* (Westwood) and two of its natural enemies: the parasitic wasp *Encarsia formosa* (Gahan) and the lady beetle *Delphastus catalinae* (Horn). Our findings revealed a strong negative correlation between increasing concentrations of karanja oil and the survival of both adult and nymphal stages of greenhouse whiteflies. In whole plant trials, the highest concentration of karanja oil solution (5% v/v%) killed 80% of the whitefly adults and 84% of nymphs, while the lowest concentration (1.25% v/v%) killed 45% of adults and 53% of nymphs. Karanja oil also exhibited a residual negative effect on immature whiteflies and did not affect the parasitism rate of *E. formosa*. The direct application of karanja oil to non-pest predators and parasitoids was lethal, indicating that in integrated pest management programs for greenhouse whitefly in tomatoes, natural enemies should be introduced after the application of botanical extracts or used exclusively without pesticides.

## 1 Introduction

Trophic webs characterize direct flows of energy through the consumption of organisms by other organisms, but do not typically consider the quality of different food items (1). In the most classical depiction of trophic webs, plants or primary producers serve as food for herbivores or primary consumers, which in turn become food for predators and parasitoids (2, 3). Trophobiotic interactions within the trophic chain are shaped by evolutionary, ecological, and environmental factors (4, 5). However, since the domestication of plants, human activity has become a primary factor that affects trophic chains. Herbivorous insects can reduce agricultural productivity (6), feeding directly from the plant and acting as vectors of viruses and plant pathogens (7), prompting the development of strategies to mitigate their impact on food crops. To that extent, pest management programs have taken advantage of the interaction between different levels of the trophic web, finding organisms that naturally feed on such herbivores.

Intensive pesticide-based management of insects has numerous ecological consequences (8) and may interfere with the effectiveness of biological control programs. Despite the ubiquity of using natural enemies in integrated pest management (IPM), there is a lack of understanding of the ecological and evolutionary complexity of the interactions between plants, herbivores, and their natural enemies. Specifically, it is unclear how plant compounds that negatively affect herbivorous pests influence the survival and performance of predators feeding on those pests. There is some evidence that toxic plant secondary metabolites can influence parasitoid performance by altering the host immune response or susceptibility (9); however, the effects of toxic metabolites on the wide range of natural enemies feeding on pest insects are relatively unknown. For the applied context, there is correspondingly little information about how insecticides, including plant-based extracts, can affect these ecological interactions across trophic levels, though some have shown that both the dose and selectivity of the insecticide determines its effects on multiple organisms within a food chain (10, 11).

Some consider botanical extracts suitable options to control insects with potentially smaller environmental effects due to their low toxicity to non-target organisms and rapid degradation (12–15). Tembo et al. (16) investigated the effects of six pesticidal plant extracts on legume crops, evaluating their effects on three different insect pests, crop yield, and beneficial arthropods. Their findings suggested that mainly extracts from three plant species (*Tephrosia vogelii, Tithonia diversifolia*, and *Lippia javanica*) effectively reduced insect pest populations and had no significant impact on beneficial arthropods compared to the negative control. Moreau et al. (17) evaluated neem oil and three traditionally used botanical extracts to control the Colorado potato beetle *Leptinotarsa decemlineata* (Say) and found that neem oil was the most effective treatment, reducing the pest population by 66% without significant non-target effects on potato plants. Similarly, seed extracts from *Crotalaria stipularia* (Desv.) have proven effective in controlling the red flour beetle *Tribolium castaneum* (Herbst), leading to 100% mortality in the insect pest, with no significant toxicity to non-target organisms tested (18).

Karanja oil, extracted from the seed of *Pongamia pinnata* L (Fabaceae), is well-known for its insecticidal effects against a wide range of insect pests (19–21). This oilseed crop is cultivated for its numerous medicinal properties (19, 22) and agricultural applications (20). The phytochemical properties of karanja oil make it valuable for integrated pest management programs (20, 22, 23). The biological activity of karanja extraction is primarily attributed to karanjin, the main flavonoid found in the seeds, which serves as the principal ingredient responsible for its lethal or deterrent effects on insects (19). Karanja oil exhibits a diverse range of effects on insect pests, including lethal, deterrent, antifeedant, and larvicidal properties against moths (24), true bugs (25), beetles (26), termites (27), mites (28), and mosquito larvae (29). However, there is limited information on how karanja oil may, directly and indirectly, affect both the adult and immature stages of *Trialeurodes vaporariorum*.

The cultivated tomato, *Solanum lycopersicum*, is commonly grown in greenhouse production systems, where it faces a range of pest issues (30) and is often a candidate for augmentative biological control, among other strategies (6). One of the most detrimental pests of tomato plants is the greenhouse whitefly, *T. vaporariorum*. Whiteflies settle on and feed from plant leaves, disrupting physiological processes, acting as virus vectors, and ultimately reducing crop yield (31, 32). Two natural enemies, the parasitoid wasp *Encarsia formosa* and the predatory beetle *Delphastus catalinae* have been tested and reported as effective against whiteflies (33, 34). *Encarsia formosa* is an endoparasitic wasp that oviposits on the immature stages of *T. vaporariorum* (33–35). The larvae feed on the internal contents of the whitefly nymphs, and functional adult parasitoids emerge after 19 to 21 days (33). *Delphastus catalinae*, a coccinellid beetle, is known for its voracity; both larvae and adults feed on *T. vaporariorum* nymphs, consuming up to 100 individuals per day (36, 37). Both of these natural enemies are commonly used in greenhouse integrated pest management (IPM) programs for biological control (38).

The main objective of this research was to evaluate the effects that karanja oil, a plant botanical extract, has on a common greenhouse food web including crop tomatoes, greenhouse whiteflies, and two commonly used biological control agents *E. formosa* and *D. catalinae.* In our study we hypothesized that the application of karanja oil would effectively kill whitefly adults and their immature stages, but with potentially deleterious effect on their predators and parasitoids depending on the timing of application. In addition, we sought to assess the suitability of using karanja oil and these natural enemies concurrently by estimating the effects of karanja oil across the lifecycle of the greenhouse whitefly.

## 2 Materials and methods

### 2.1 Plant material

For all experiments, we used tomato plants (*Solanum lycopersicum*, cv. Moneymaker), a common host for greenhouse whiteflies, and cultivated them under controlled conditions (temperature: 22–25 °C, relative humidity: 70–80%, 16:8 light/dark cycle) in the Horticulture Greenhouse at the University of British Columbia (Vancouver, British Columbia, Canada).

To break dormancy, we soaked the tomato seeds in fresh water for 12 hours, then placed them on a moist Petri dish in the dark for 8 hours to stimulate embryo growth and elongation. Then, we sowed the seeds at a depth of 1.5 cm in 1.5-liter pots filled with soil mix and kept them on a high-humidity seedling bench for four weeks. After this period, we transferred the plants to greenhouse benches with flood irrigation.

Throughout the seedling and experimental stages, we irrigated the plants exclusively with fresh water. To prevent any potential stimulation of plant growth or interference with the insect infestation process, we did not apply fertilizers, plant hormones, or growth promoters. We conducted all experiments using 7- to 8-week-old plants.

### 2.2 Insects

For every trial performed with herbivore insects, we introduced same-aged (5 weeks old) whitefly adults *T. vaporariorum* (Hemiptera: Aleyrodidae, greenhouse whitefly) to observation enclosures (Rearing & Observation Cage, 34.3 x 34.3 x 61 cm- from BioQuip, California, USA). To supply whiteflies, we followed the methodology detailed by Polston and Capobianco (39). We reared whiteflies in colonies under controlled conditions (temperature: 22 – 25 °C, relative humidity: 70 to 80%, 16:8 light/dark cycle) and maintained the colony by regularly adding whiteflies to 6 to 7-week-old, insect-free tomato plants (cv. Amish Paste). We collected new adults after 17 to 20 days.

We obtained the parasitoid wasps from Koppert Biological Systems (Scarborough, Canada) with a product called *En-strip*^®^. This product contains cardboard units (3 cm x 2 cm) with 40 whitefly nymphs parasitized by *E. formosa.* Adult emergence generally occurs after two weeks. Once the adult wasps emerge, they are capable of parasitizing new whitefly nymphs. Additionally, we obtained adult predatory beetles from Koppert Biological Systems (Scarborough, Canada) using a product named *Delphibug*^®^. After unpacking, adult beetles in buckwheat hulls were immediately ready to be introduced into the experimental units.

### 2.3 Karanja (*P. pinnata*) oil

The karanja oil formulation was supplied by Terramera Inc. (Vancouver, British Columbia), consisting of 85% pure karanja oil and 15% inert emulsifier. This plant extraction was not a commercial formulation, and we used it exclusively for investigative purposes only. In our trials, we utilized 100% pure, cold-pressed karanja oil, this extraction method has been shown to yield a karanjin concentration of approximately 2,000 ppm (40–42). We obtained the test concentrations by warming the formulations in a hot water bath at 30 °C for 15 to 20 minutes and then diluting them to experimental concentrations with distilled water.

### 2.4 Insect bioassays

We conducted a series of insect bioassays to assess the concentration-dependent effects of karanja (*P. pinnata)* extracts on *T. vaporariorum*. Initially, we tested the immediate effects of karanja oil on adult survival, followed by longer-term effects on both adult survival and reproduction rates. A third experiment evaluated the impact of directly spraying karanja oil on nymphs, while a final trial (See **Supplementary Material**, Application of karanja (*P. pinnata)* oil to the plant root system) assessed the potential systemic effects of a karanja oil solution.

Additionally, we performed four bioassays to examine the effects of karanja oil within a tri-trophic interaction framework. First, we conducted two experiments to determine whether the direct application of karanja oil affected the survival of the parasitoid wasp *E. formosa* or the predatory beetle *D. catalinae*. Second, we carried out two experiments to investigate the potential effects of prey treated with karanja oil on the performance of natural enemies.

We conducted all experiments under controlled laboratory conditions at the Horticultural Greenhouse at the University of British Columbia (Vancouver, British Columbia, Canada, unceded x^w^məθk^w^əy̓əm Musqueam Territory).

### 2.5 Karanja (*P. pinnata)* oil phytotoxicity on tomato plants (*S. lycopersicum*)

Botanical extracts can impact the growth and development of plants (43). Therefore, we evaluated the potential phytotoxic effects of karanja oil on tomato plants by registering any leaf discoloration, chlorosis, necrosis, or leaf deformation in all trials.

### 2.6 The effect of karanja (*P. pinnata)* oil on the whitefly lifecycle

To evaluate both the immediate lethal effects and longer-term impacts on offspring development and surviving adults, we sprayed different concentrations of karanja oil solutions (1.25%, 2.5%, and 5% (v/v%)) as well as a pure water control on 7-week-old tomato plants (*S. lycopersicum*) until they were fully covered. We applied 4 ml of solution per plant. Once the solution had dried, we placed the treated tomato plants and falcon tubes containing the same-aged whitefly adults (n=20 for each experimental unit) into micro-perforated plastic bags to allow for natural gas exchange while preventing the escape of the whiteflies, with ten replicates for each treatment.

The experiment consisted of two stages. Initially, we counted the number of surviving adults 48 hours after applying karanja oil. Two weeks after the application, we recorded the number of adults again, as well as the number of nymphs and eggs, differentiating them according to their instar levels.

### 2.7 The effect of karanja (*P. pinnata)* oil on *T. vaporariorum* offspring survival

This experiment had two objectives: first, to evaluate the effect of karanja oil solution when applied directly to *T. vaporariorum* nymphs, and second, to assess whether the emulsifier had a lethal effect on the nymphs. We conducted a bioassay by introducing same-aged whitefly adults into enclosures containing 7-week-old tomato plants (*S. lycopersicum*). We grew and maintained the tomato plants as described above and then placed them in mesh cages (Rearing & Observation Cage, 34.3 x 34.3 x 61 cm, Bioquip, California, USA) (Illustration 1. **Supplementary Material**). Afterward, we released same-aged whitefly adults (n=40 for each experimental unit) to initiate the infestation process, using five replicates for each treatment.

Two weeks after whitefly inoculation, we extracted the adults and applied a karanja oil solution (1%, 2%, or 3% (v/v%)), a water control, or an emulsifier control directly onto the nymphs, at an application rate of 4 ml of treatment per plant. We counted the total number of nymphs before applying the treatments and recorded the number of surviving nymphs 48 hours after we applied the treatments.

### 2.8 The effect of karanja (*P. pinnata)* oil on *Encarsia formosa*

We assessed the effect of the direct application of karanja oil on developing parasitoid wasps, introducing a cardboard unit containing 40 whitefly nymphs parasitized by *E. formosa* into a Petri dish. Next, we applied different concentrations of karanja oil solutions (0.5%, 1%, 2%, or 3% (v/v%)) or a water control directly to the parasitized nymphs adhered to the cardboard unit, ensuring they were completely covered with the solution. We used 11 replicates for each treatment and counted the number of emerging adults two weeks after the application.

### 2.9 The effect of karanja (*P. pinnata)* oil on *Delphastus catalinae*

To evaluate the effect of the direct application of karanja oil on the predator beetle *D. catalinae*, we introduced ten adults into each Petri dish and sprayed them directly with different concentrations of karanja oil solutions (0.5%, 1%, 2%, or 3% (v/v%)) or with water control. We used 11 replicates for each treatment and counted the number of surviving adults 24 hours after the application of the treatment.

### 2.10 The effect of karanja (*P. pinnata)* oil on the parasitism performance by *Encarsia formosa*

The main objective of this experiment was to evaluate the effect of karanja oil on the parasitism performance of *E. formosa* on *T. vaporariorum* nymphs. Initially, we sprayed seven-week-old tomato plants (*S. lycopersicum*) with different concentrations of karanja oil (0.5%, 1%, 2%, or 3% (v/v%)), as well as water control or an emulsifier control, using an application rate of 4 ml of solution per plant. After the solution dried, we placed the tomato plants in sealed enclosures (Rearing & Observation Cage, 34.3 x 34.3 x 61 cm, Bioquip, California, USA) and introduced a falcon tube containing same-aged whitefly adults (n=40) for three weeks.

Next, we introduced a cardboard unit with 40 whitefly nymphs parasitized by *E. formosa* from Koppert Biological Systems (Scarborough, Canada). We estimated the number of parasitoid wasps needed for the trial based on the number of whitefly adults released and their potential fecundity. According to Perring et al. (32) and Lloyd (44), a single whitefly can lay up to 110 eggs in its adult stage. Introducing 40 parasitoids would cover the potential offspring of the whiteflies over two weeks, as a single wasp can parasitize eight nymphs per day (33). Further, *Encarsia formosa* is well known for its ability to determine whether a nymph is parasitized, allowing it to avoid both non-self and self-super parasitism with remarkable efficiency (33).

We used five replicates for each treatment and counted the number of parasitized nymphs and the total number of surviving nymphs per experimental unit two weeks after introducing the parasitic wasps.

### 2.11 The effect of karanja (*P. pinnata)* oil on the predatory performance by *Delphastus catalinae*

We evaluated the effect of karanja oil on the predatory performance of *D. catalinae* on *T. vaporariorum* nymphs. The general setup for this experiment, including the tomato plants and karanja oil concentrations, was identical to that used in the *E. formosa* trials. However, for this experiment, we added an additional treatment as an absolute control, where we did not apply karanja oil nor released predator beetles. This positive control was included because *D. catalinae* beetles consume the entire nymph. In contrast, such a situation did not occur in the *E. formosa* trials, where we were able to count the number of parasitized nymphs.

We released 10 *D. catalinae* adults for all experimental units and used five replicates for each treatment. We counted the total number of whitefly nymphs per experimental unit two weeks after the release of the predatory beetles.

### 2.12 Statistical analysis

All experiments utilized complete randomized experimental designs. We transformed and normalized data when needed for all experiments except for the survival of natural enemies. Afterwards, we used a linear model (LM) and performed an ANOVA with karanja oil concentration as a fixed factor to test the differences among treatment levels. We carried out a Tukey *post-hoc* test to identify the differences between treatments with a significance level 0.05. For the survival of natural enemies bioassays, we performed a Kruskal-Wallis nonparametric test to assess the differences among treatment levels, and we utilized a *post-hoc* Wilcoxon test with Bonferroni correction to identify the differences between treatments. We used a significance level of 0.05 for all analyses.

For all trials, we counted the number of surviving whitefly adults and the number of nymphs alive with karanja oil concentration as both a categorical and an explanatory factor. We analyzed the number of surviving nymphs with karanja oil concentration as an explanatory factor for the offspring survival experiment. For analyzing the parasitoid and predator performance, we counted the number of surviving whitefly nymphs (and the number of nymphs parasitized) with karanja oil concentration as both a categorical and an explanatory factor. Finally, for the natural enemy survival experiments, we analyzed the number of surviving wasps and beetle nymphs with karanja oil concentration (as a categorical factor) as an explanatory factor.

We performed our analyzes in R software version 4.5.0 (45), and our figures were drawn using ggplo2 (46). The artwork and illustrations were drawn with Adobe Illustrator (47).

## 3 Results

### 3.1 Karanja (*P. pinnata)* oil phytotoxicity on tomato plants (*S. lycopersicum)*

Karanja oil did not have phytotoxic effects on tomato plants at the concentrations we applied. Across the series of experiments, we did not observe any instances of discoloration, chlorosis, necrosis or leaf deformation in response to karanja oil.

### 3.2 Effects of karanja (*P. pinnata)* oil application on *T. vaporariorum* adults

#### 3.2.1 Adult survival

**Figure 1:**
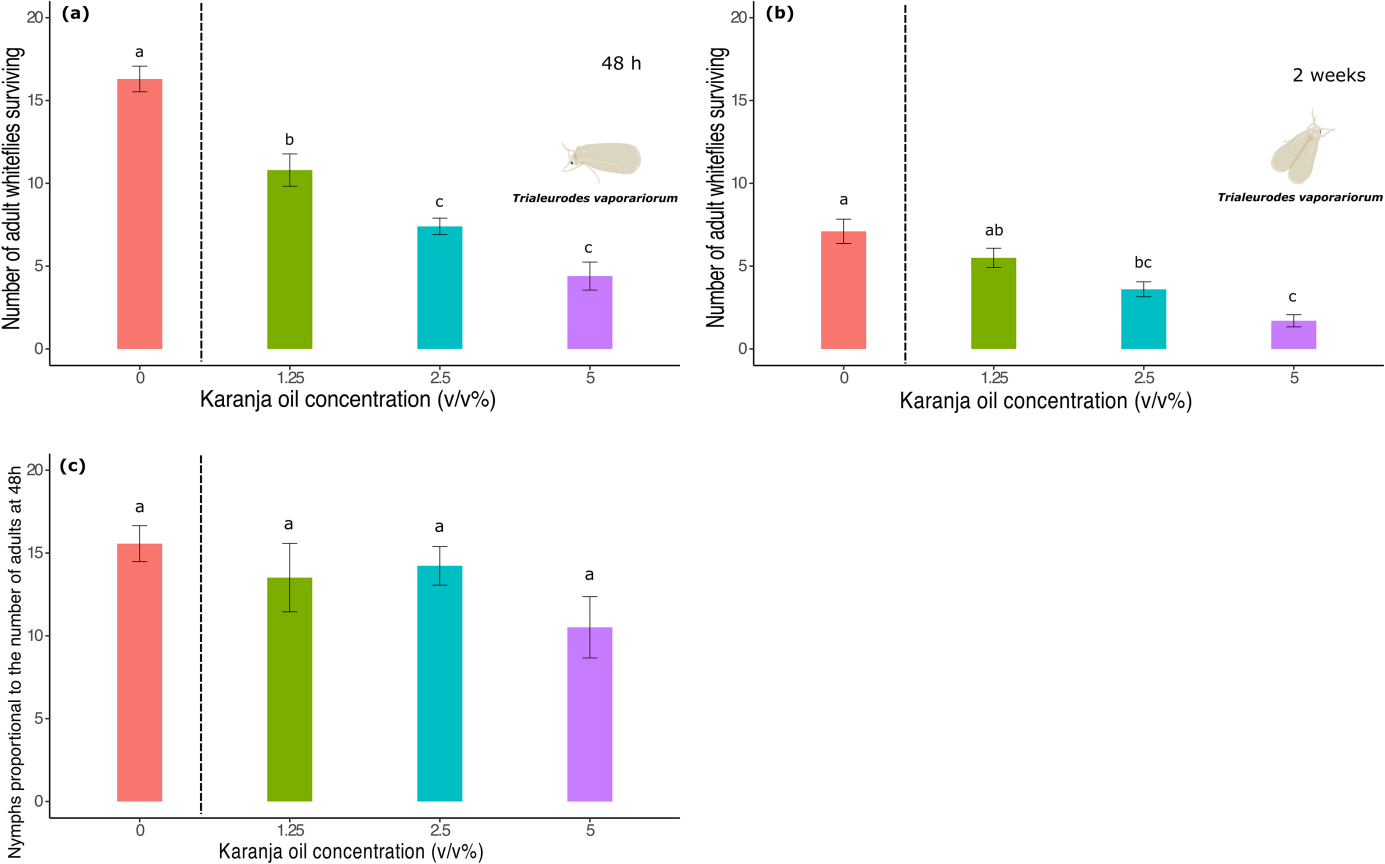
Number of *T. vaporariorum* adults surviving on plants sprayed with different concentrations of karanja oil (Control, 0; 1.25%; 2.5%; 5%). **(a)** 48 hours after the application event. **(b)** Two weeks after the application event. **(c)** Number of nymphs per whitefly. Means and standard error shown. Different letters indicate significant differences.

After 48 hours of treatment application, the number of living adult *T. vaporariorum* decreased following exposure to karanja oil (karanja: F3,36 = 41.40, p < 0.0001; **Figure 1**). Compared to the control treatment, where 20% of whitefly adults died, applying karanja oil significantly reduced the number of whitefly adults. Such an effect was dependent on the concentration. Specifically, a karanja oil concentration of 5% killed 80% of the whitefly adults, whereas a karanja oil concentration of 2.5% and 1.25% killed 65% and 45% of the *T. vaporariorum* adults, respectively (**Figure 1a**). Our results in this experiment show that the number of dead adults increased significantly as the concentration of karanja oil increased from 1.25% to 2.5% (p < 0.05) and 1.25% to 5% (p <0.05). Our results did not show significant differences between karanja treatments 2.5% and 5% (p > 0.05).

Two weeks after the application event, the number of *T. vaporariorum* decreased further, depending on the concentration of karanja oil (karanja: F3,36 = 17.91, p < 0.0001; **Figure 1b**). Our results suggest significant differences in the number of adult whiteflies that died between the karanja oil treatments and the control. From the initial number of adult whiteflies introduced after the application event, 75% died at a 1.25% concentration, 85% died at a 2.5% concentration, and 95% died at a 5% concentration (**Figure 1b**). However, we did not find significant differences in the number of nymphs per adult among all treatments (karanja: F3,36 = 1.77, p = 0.16; **Figure 1c**).

#### 3.2.2 Nymph survival

**Figure 2:**
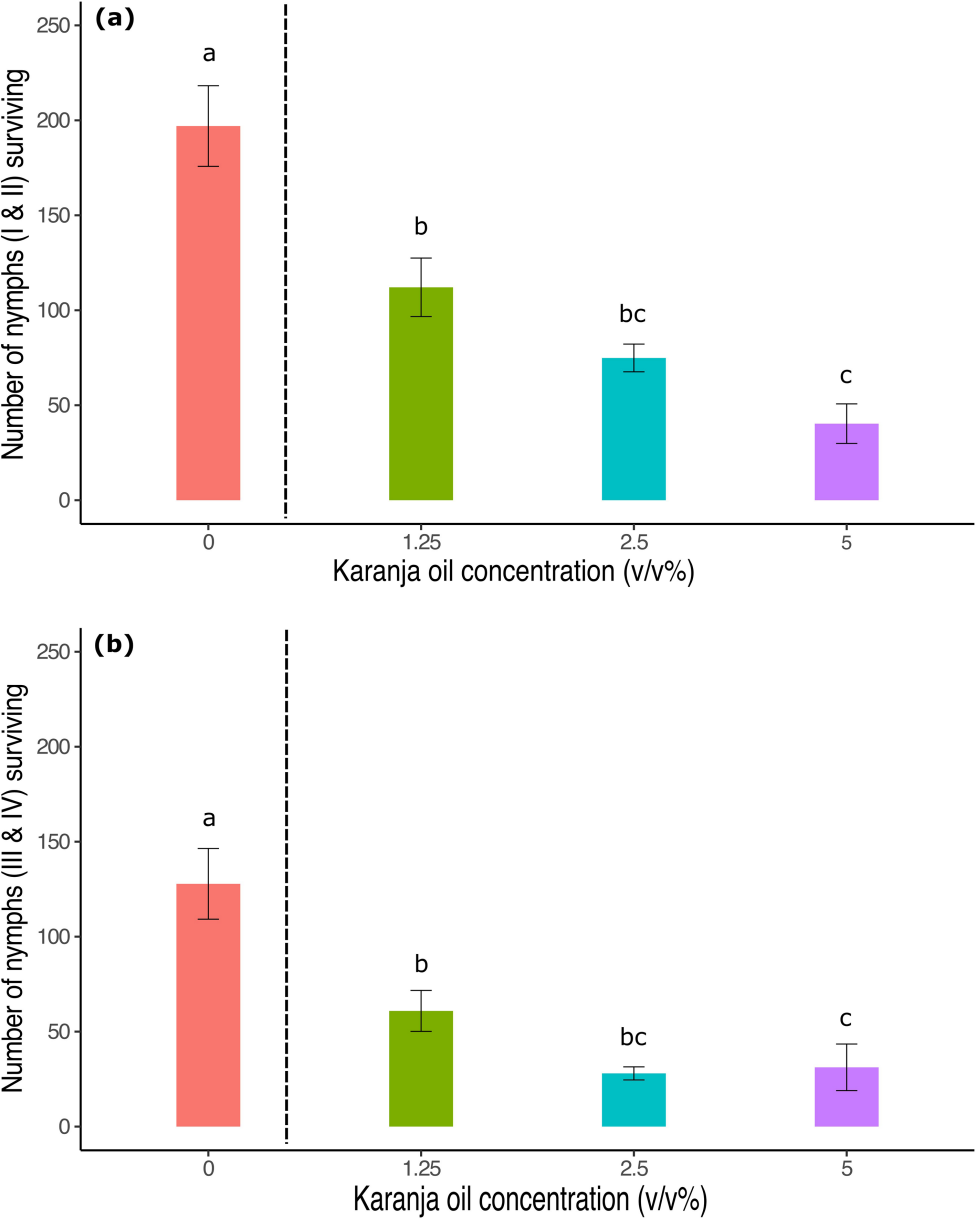
Number of *T. vaporariorum* nymphs surviving on plants sprayed with different concentrations of karanja oil (Control, 0; 1.25%; 2.5%; 5%). **(a)** Nymphal stages I and II, **(b)** Nymphal stages III & IV. Means and standard error shown. Bars with different letters are significantly different from each other.

The number of *T. vaporariorum* nymphs decreased after contact with karanja oil in a dose-dependent manner (karanja: F3,36 = 33.62, p < 0.0001; **Figure 2a**). Similarly to what occurred to the adult whiteflies in the same trial, the number of first and second-instar nymphs was significantly lower compared to the control treatment, decreasing as the concentration of karanja oil increased: 53.4%, 69.5%, and 83.6% of the nymphs died at the 1.25%, 2.5%, and 5% treatments, respectively. However, we did not find a significant difference in adult survival between the 1.25% and 2.5% karanja oil concentrations (p< 0.05).

For third and fourth instar nymphs (karanja: F3,36 = 13.29, p < 0.0001; **Figure 2b**), we found that their number also decreased significantly with increasing karanja oil concentration. Compared to the control, 76% of the nymphs died at the 5% treatment, while 78% and 53% died at the 2.5% and 1.25% treatments, respectively.

### 3.3 The effect of karanja (*P. pinnata)* oil on *T. vaporariorum* offspring survival

**Figure 3:**
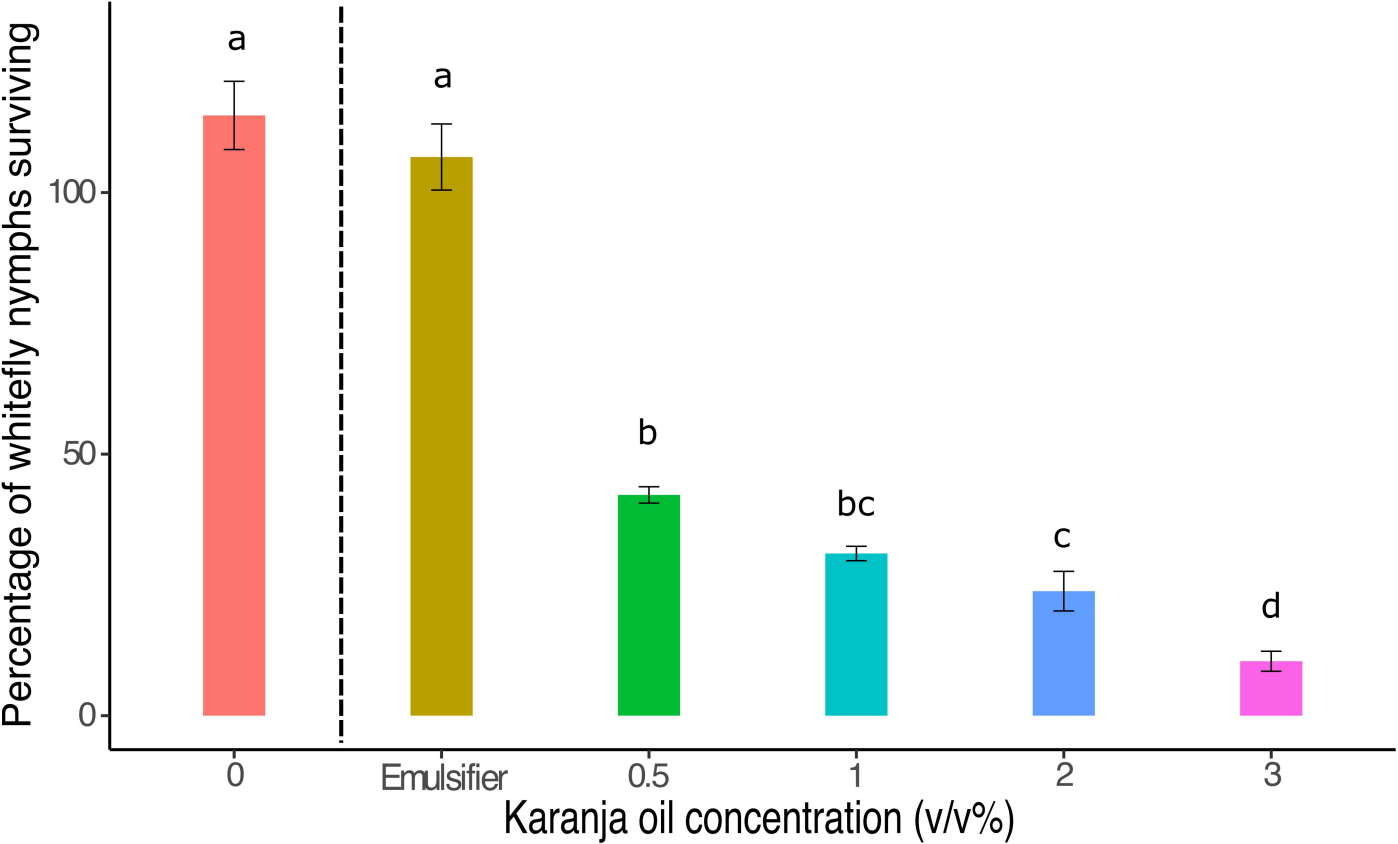
Percentage of *T. vaporariorum* nymphs surviving a direct application of different concentrations of karanja oil. With Control (0), inert emulsifier (Emulsifier), and karanja oil (0.5%; 1%; 2%; 3%). Means and standard error shown. Bars with different letters are significantly different from each other.

Karanja oil was lethal to whitefly nymphs when applied directly (karanja: F5,24 = 131.7, p<0.0001; **Figure 3**) as the number of whitefly nymphs was lower on karanja oil treatment plants compared to both the control plants and the plants treated with the inert emulsifier. In contrast, the inert emulsifier alone did not have a lethal effect on the nymphs as it did not show significant differences with the control treatment (p >0.05). The number of dead whitefly nymphs increased with a higher concentration of karanja oil, with some overlap across treatments (**Figure 3**).

### 3.4 The effect of karanja (*P. pinnata)* oil on *Encarsia formosa*

**Figure 4:**
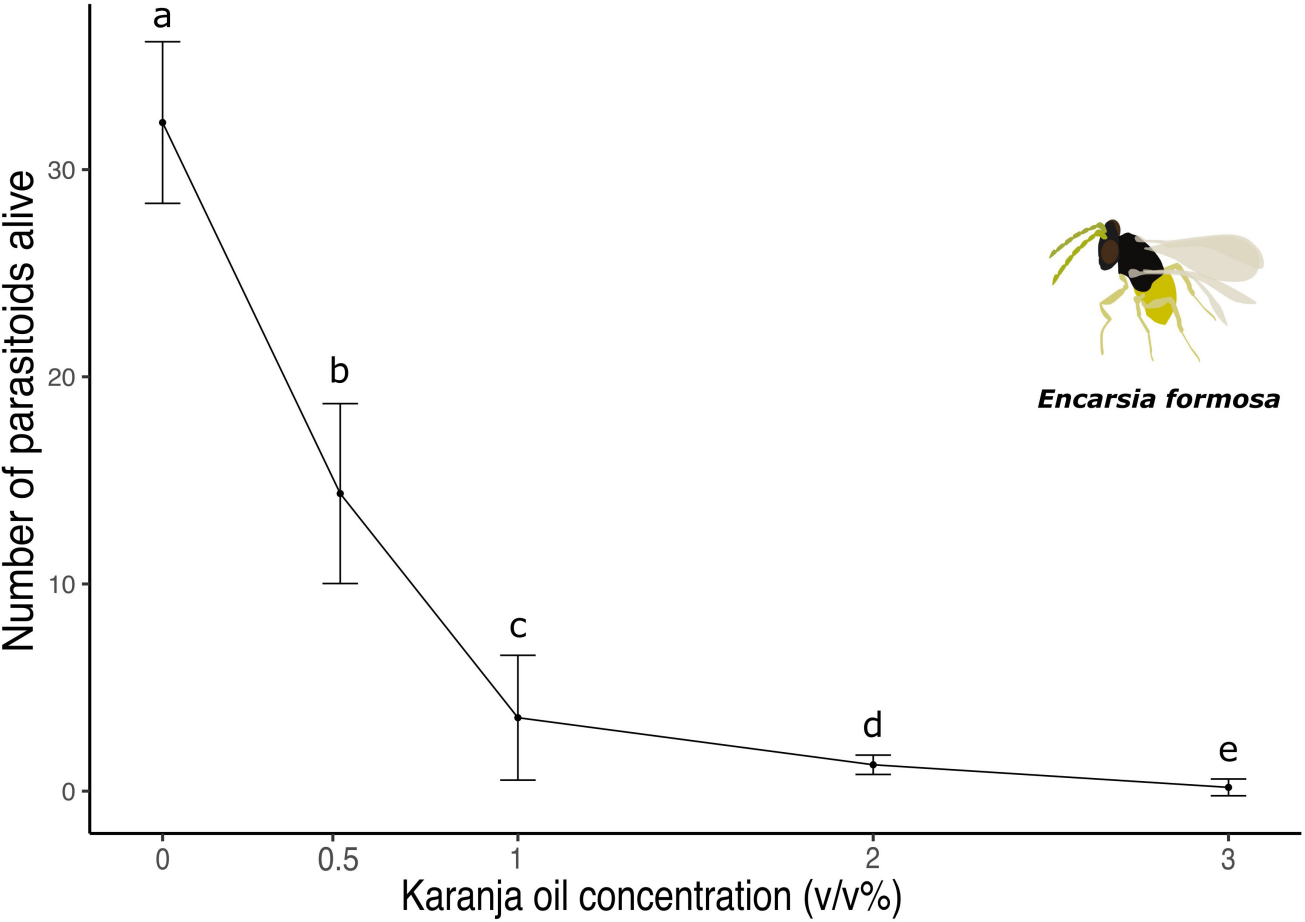
Number of *E. formosa* parasitoids wasps alive after being sprayed with different concentrations of karanja oil (Control, 0; 0.5%; 1%; 2%; 3%). Means and standard error shown. Different letters indicate significant differences.

The number of adult wasps that emerged from whitefly nymphs was significantly affected by the application of karanja oil (X²(5) = 50.41, p < 0.0001; **Figure 4**). Compared to the control, where 19.4% of adults did not emerge, applying karanja oil to parasitized nymphs significantly reduced the number of emerged individuals. At a karanja oil concentration of 0.5%, 64.1% of *E. formosa* adults died, while 91.4% and 96.8% of adults died at concentrations of 1% and 2%, respectively. We observed complete mortality in karanja oil at 3% since no adults emerged.

### 3.5 The effect of karanja (*P. pinnata)* oil on *Delphastus catalinae*

**Figure 5:**
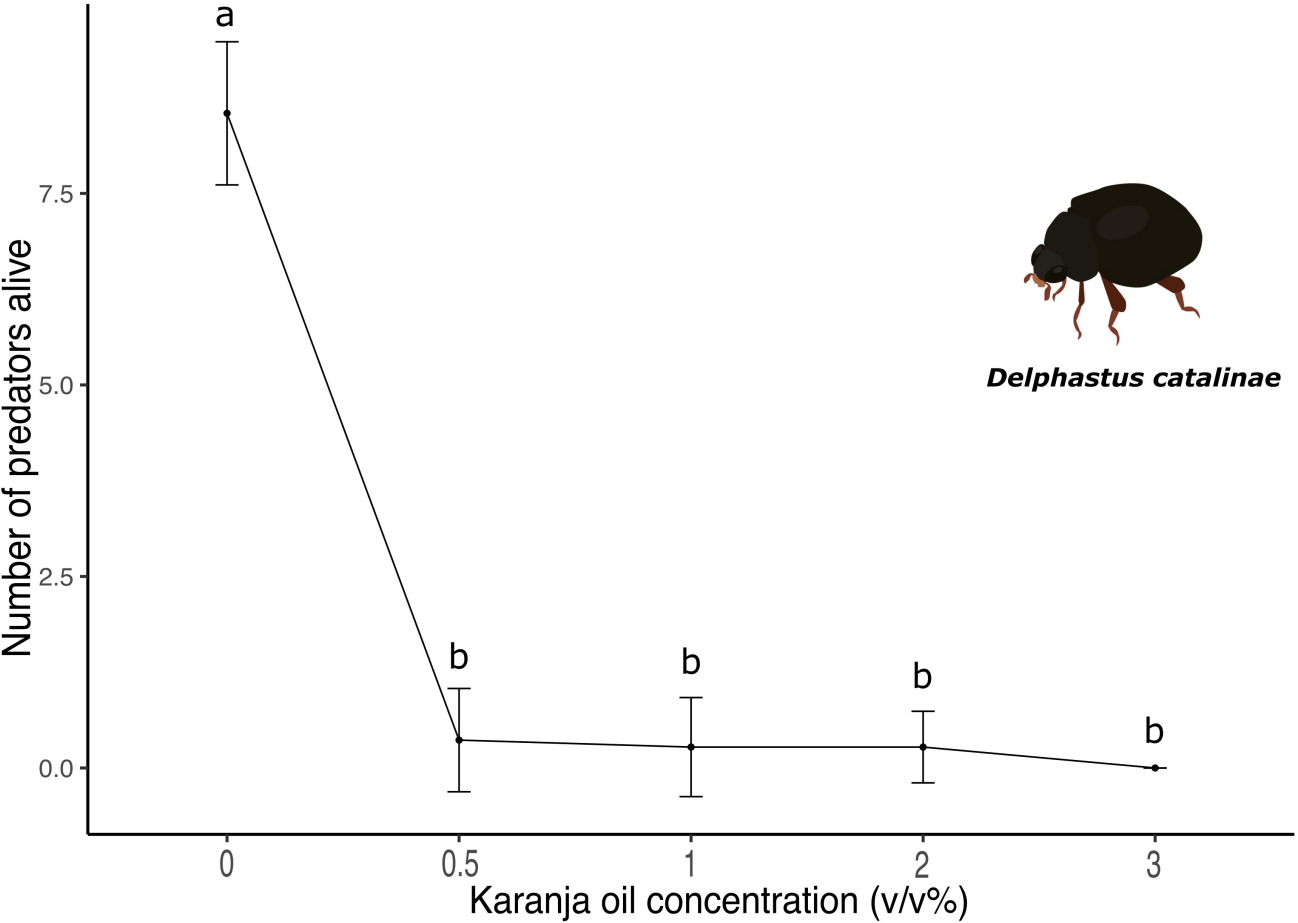
Number of *D. catalinae* predator beetles alive after being sprayed with different concentrations of karanja oil (Control, 0; 0.5%; 1%; 2%; 3%). Means and standard error shown. Different letters indicate significant differences.

Karanja oil killed *D. catalinae* adults 48 hours after its application (X^2^ (5)=37.53, p<0.0001; **Figure 5**). Regardless of the concentration, we observed a significant decrease in the number of surviving adults in all treatments compared to the control. Due to the strong lethal effect, we did not find significant differences between karanja oil treatments (p < 0.05). Specifically, 7 out of 10 adults died at karanja 0.5%, 1%, and 2%, whereas all individuals died at karanja 3%.

### 3.6 The effect of karanja (*P. pinnata)* oil on the parasitism performance by *Encarsia Formosa*

**Figure 6:**
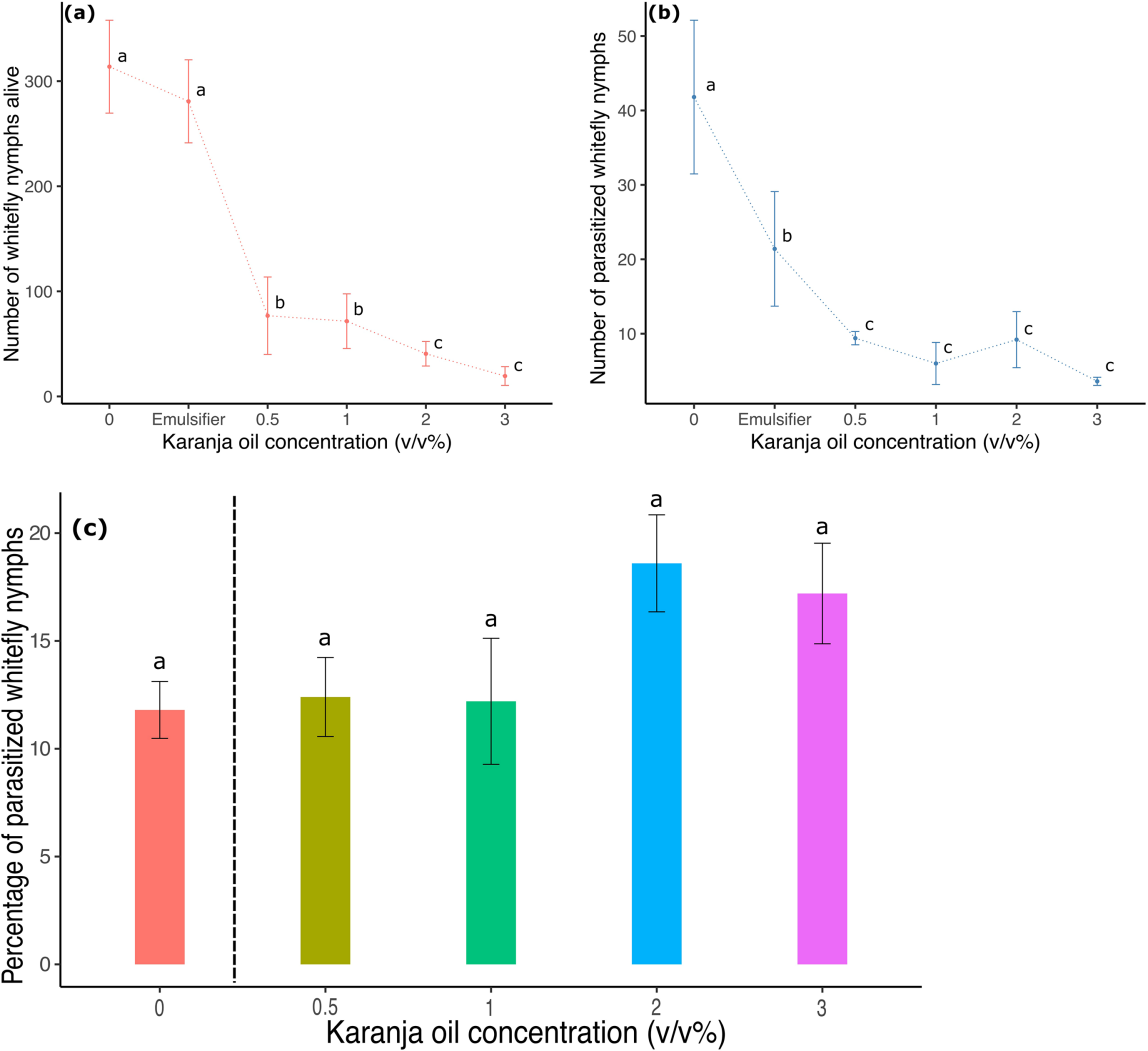
Number of *T. vaporariorum* nymphs on plants sprayed with different concentrations of karanja oil (Control, 0; Emulsifier; 0.5%; 1%; 2%; 3%) before the introduction of *E*. *formosa*. **(a)** Whitefly nymphs alive. **(b)** Parasitized whitefly nymphs, and **(c)** Percentage of parasitized whitefly nymphs. Means and standard error shown. Different letters indicate significant differences.

Karanja oil did not affect the relative parasitism rate of *E. formosa* on *T. vaporariorum* nymphs. The number of nymphs alive decreased and was significantly different between control and karanja oil treatments (karanja: F5,24 = 51.73, p<0.0001; **Figure 6a**), but the parasitism rate was not affected (**Figure 6b**). Compared with the control in which 12% of nymphs were parasitized, there were 17.2% of nymphs parasitized at karanja 3%, whereas 18.6%, 12.2%, and 12.4% of immature insects were parasitized at karanja 2%, 1%, and 0.5%, respectively (**Figure 6c**). Our results show that there were no significant differences in the percentage of nymphs parasitized between karanja oil treatments.

### 3.7 The effect of karanja (*P. pinnata)* oil on the predatory performance by *Delphastus catalinae*

**Figure 7:**
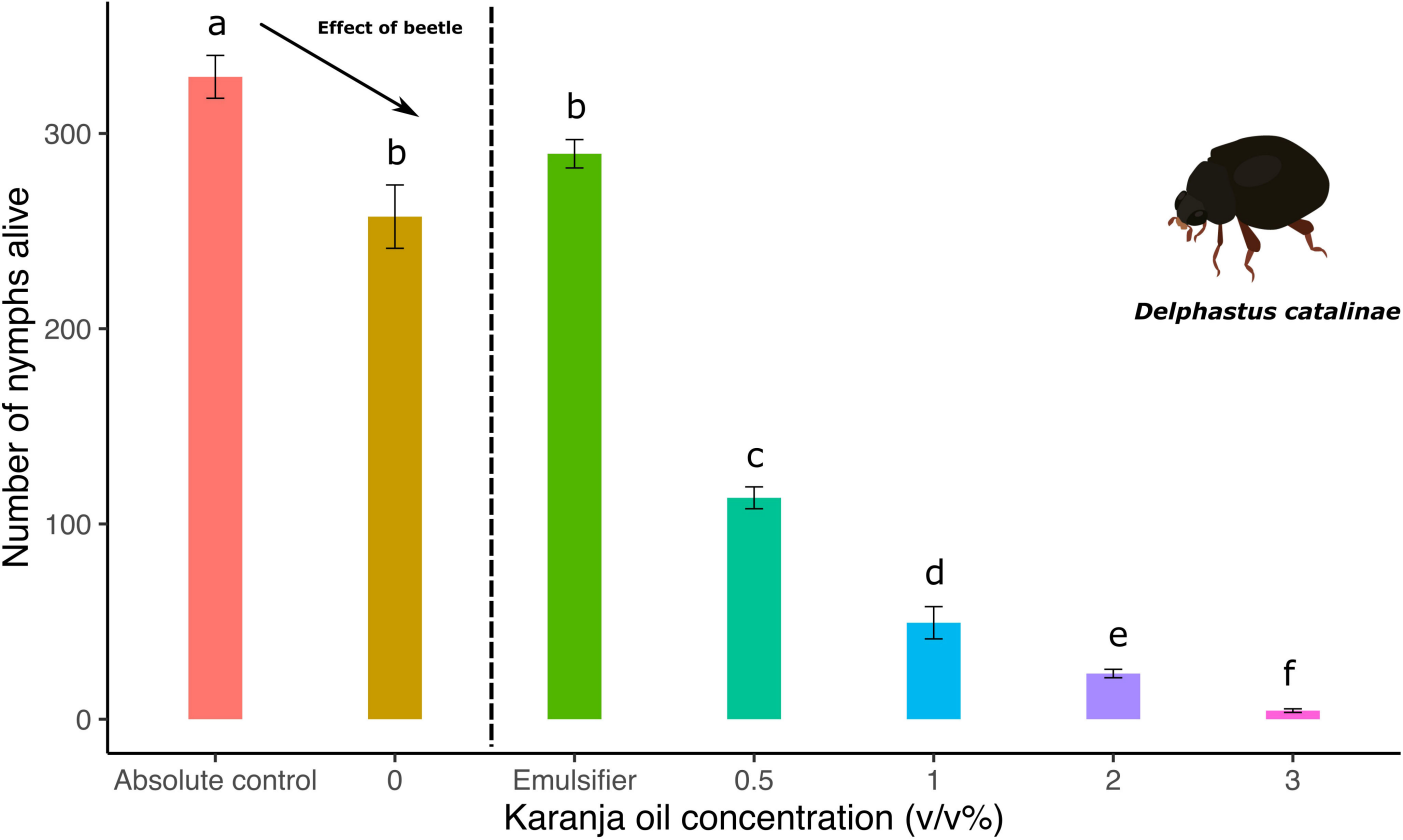
Number of *T. vaporariorum* nymphs on plants sprayed with different concentrations of karanja oil (Absolute control (no karanja application and no predators released); Water Control, 0; Emulsifier; 0.5%; 1%; 2%; 3%) before the introduction of *D. catalinae*. Means and standard error shown. Different letters indicate significant differences.

The combined effect of karanja oil application and the release of *D. catalinae* predators effectively reduced the number of whitefly nymphs (karanja: F6,28 = 284.24, p<0.0001; **Figure 7**). Compared to the water control, our results suggest that the number of dead nymphs increased significantly in karanja oil treatments with predator beetles. After the release of the predators, we found that 55.9% of *T. vaporariorum* nymphs died at karanja 0.5%, whereas 80.8% and 90.9% died at karanja 1% and 2%, respectively. In this trial, the combined effect of karanja oil at 3% and the release of predator beetles had a strong effect on the number of nymphs since 98.3% of immature whiteflies died. Finally, we did not find significant differences between water and emulsifier control (p > 0.05).

## 4 Discussion

After evaluating the trophic interactions between tomato plants, white flies, predator beetles, and parasitoid wasps and assessing how such interactions change when mediated by the application of a botanical extract, our results strongly support that karanja oil was detrimental to the survival of every level of the trophic web except tomato plants, but that karanja oil does not necessarily affect the interaction between *T. vaporariorum* and their natural enemies:

In the first place, the lethal effect of karanja oil on adult whiteflies suggests that this effect is correlated with the concentration of karanja oil (**Figures 1**, **3**). The toxic effects of karanja oil persisted for up to two weeks after application under greenhouse conditions, reducing the total number of adults (**Figure 1b**) and nymphs (**Figure 2**) compared to untreated plants. However, our results indicate that the reduction in the number of whitefly nymphs is not associated with a decrease in the number of eggs laid by adults (**Figure 1c**), as we did not observe significant differences in the number of nymphs per adult across all treatments. This suggests that the decrease in the number of nymphs is primarily due to the reduction in the survival of adult whiteflies.

Some researchers have studied the lethal effects of karanja oil on various agricultural insect pests. Ghosal (48) investigated the lethal impact of karanja oil on *T. vaporariorum* and found that the effects of karanja oil persisted on adult whiteflies for at least five days after spraying. Other studies further support the insecticidal properties of karanja oil on both adult and immature stages of insect pests, suggesting that it is lethal by contact and that its effects persist for several days (19, 49, 50).

Once we determined that karanja oil had a lethal effect on adult whiteflies and that this effect persisted over time, it was necessary to investigate whether the reduction in the number of offspring was solely due to the decrease in adult survival or if karanja oil also affected whitefly nymphs through direct contact. Our results suggest that karanja oil kills the immature stages of *T. vaporariorum* when applied directly (**Figure 3**), and the number of dead nymphs is correlated with the concentration of this botanical extract (**Figure 2**, **Figure 3**). These findings are consistent with previous research that has evaluated the lethal effects of karanja oil on the immature stages of other insect pests. For example, Hiremath et al. (51) reported a 95% lethal effect when karanja oil was applied directly to the immature stages of the brown planthopper (*Nilaparvata lugens* Stal). Similarly, karanja oil has shown lethal effects on the offspring of other insect pests, including the Colorado potato beetle (*Leptinotarsa decemlineata* Say) (49) and the diamondback moth (*Plutella xylostella* L.) (52).

Our results are supported by a set of bioassays that suggest that karanja oil is potentially suitable as a biopesticide of whiteflies in IPM programs. Ghosal (48) found that a karanja oil solution of 2 ml/l applied to whitefly adults on tomato plants killed 56.5% of the insect population after 20 days. These results were consistent in a replicate conducted the following season, where 54.5% of adult whiteflies died due to the lethal effect of karanja oil. Ghosal (48) concluded that the lethal effect might be augmented if the concentration increases, and to this extent, our results support that statement. In research conducted over two seasons, Kumar et al. (53) found that a concentration of 1% of karanja oil reduced the population of whiteflies in a cotton crop by 58.5% in the first season and 51.7% in the second. Similar results were reported by Ghosal (48) in cotton crops. In our study, a lower concentration of karanja oil (2ml/l) killed 53.4% of the whitefly population.

**Figure 8:**
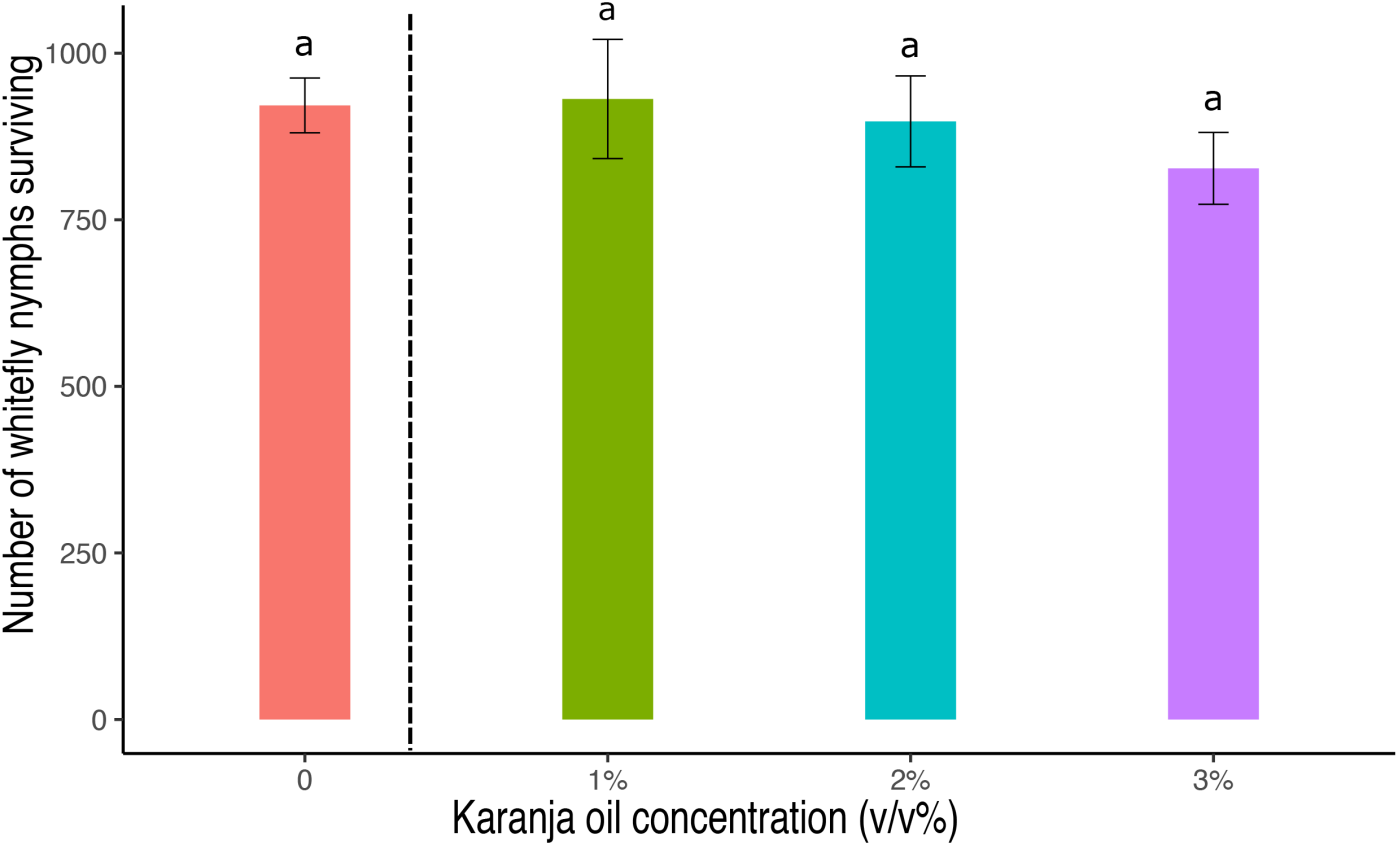
Number of *T. vaporariorum* nymphs surviving after 3 weeks of pouring different concentrations of karanja oil to the roots (Control, 0; 1%; 2%; 3%). Means and standard error shown. Different letters indicate significant differences.

Regarding the mode of action, our results indicate that karanja oil exerts a toxic effect through direct contact with *T. vaporariorum* adults and immature stages (**Figures 1**, **3**). However, it is important to note that we do not have sufficient evidence to rule out the possibility of a systemic action, although the lack of insecticidal effect with root application of karanja oil would suggest that systemic effects may be minimal (See **Supplementary Material**, **Figure 8**). Plant tissue analysis would be necessary to clarify this aspect. The contact mode of action of karanja oil is supported by research conducted by Lengai et al. (21) as well as studies by other authors (19, 50, 52, 54), all of which suggest that the primary lethal effect of karanja oil on insect pests occurs when the oil directly contacts the insect or when insects come into contact with treated surfaces.

As to natural enemies and their interaction with karanja oil, our results show that karanja oil is compatible with the natural enemies of *T. vaporariorum* as long as it does not come into direct contact with them. While we confirmed that karanja oil reduced the survival of *T. vaporariorum* offspring (**Figure 6a**), we found that the proportion of parasitism by *E. formosa*—measured as the relative rate of parasitism to the number of living nymphs—was not significantly impacted by the application of karanja oil (**Figure 6b**). However, our findings also indicate that karanja oil does not enhance parasitism rates, suggesting that it does not increase the susceptibility of *T. vaporariorum* to parasitism.

Although limited research has specifically examined the tri-trophic levels considered in this study, our findings align with other studies that have evaluated the effects of karanja oil and other botanical extracts on various pests and parasitoids. For instance, Soosaimanickam and Savarimuthu (55) suggested that karanja oil is compatible with the endoparasitic wasp *Trichogramma chilonis* (Ishii). In their study, concentrations of karanja oil (0.15%, 0.3%, and 0.5%) did not affect parasitoid emergence compared to the control, while the solution applied effectively killed 46.6% of the insect pest *Spodoptera litura* (Fabricius). Similarly, Ateyyat et al. (56) reported that flavonoids—major components of karanja oil—had minimal impact on the interaction between the woolly apple aphid *Eriosoma lanigerum* (Hausmann) and its endoparasitoid *Aphelinus mali* (Hald). In their study, the highest concentration tested (1%) did not affect parasitoid emergence compared to the control.

A common finding in these studies, consistent with our results, is that botanical extracts do not increase parasitism rates. Our data suggest that the performance of *E. formosa* remains unaffected by karanja oil concentration, regardless of whether the solution is applied before the release of parasitoids. This observation supports the findings of the referenced studies.

Our results suggest that the combination of karanja oil and the release of predators reduces the number of *T. vaporariorum* nymphs alive (**Figure 7**). However, the absence of positive controls in a fully crossed design makes it unclear whether karanja oil directly interferes with or enhances the predatory performance of *D. catalinae*. Since *D. catalinae* is an active predator of *T. vaporariorum* nymphs (36, 37), it is commonly used in integrated pest management (IPM) programs. In some cases, the release of predators is combined with the application of botanical extracts, and several studies have investigated the potential effects of such combinations on predator beetle populations.

For instance, Ghosal (48) evaluated the effect of a 0.2% concentration of karanja oil on coccinellid beetles, which prey on whiteflies. He found that karanja oil was not lethal to predator beetles, as their numbers did not differ from those in the control treatment. Similar findings were reported by Sahana and Tayde (57) in a study where they applied 3% karanja oil against the fruit borer *Leucinodes orbitalis* (Guenée). They observed no significant change in coccinellid beetle populations seven days after spraying. Additional studies by Sakthivel et al. (58) and Mukhopadhyay et al. (59) also found that 2 and 3% concentrations of karanja oil were highly effective in controlling various insect pests without negatively affecting coccinellid beetle populations.

Finally, our findings reveal that karanja oil is highly lethal to *E. formosa* and *D. catalinae* when applied directly (**Figures 4**, **5**). While limited research has specifically examined the effects of karanja oil on natural enemies, there is substantial evidence that bioinsecticides can have non-target effects on the natural enemies of insect pests, including predators and parasitoids that provide valuable ecosystem services. Although these effects depend on factors such as the concentration of botanical extracts, mode of action, and active ingredients, studies have shown that impacts can range from reduced adult survival to decreased reproductive success (60).

Our results are consistent with studies in which botanical extracts were applied directly to predators and parasitoids. For example, Simmonds et al. (61) found that a 100 ppm concentration of neem extracts significantly reduced the adult emergence of *E. formosa* from parasitized *T. vaporariorum* nymphs, with the nymphs submerged in the test solution. Other studies have shown that the adult emergence of ichneumonid endoparasitoids decreases when their host nymphs are exposed to neem extracts (62). The toxic effects of botanical extracts on predator beetles have also been investigated. Mollah et al. (63) found that a 10% neem solution was lethal to the coccinellid beetle *Adonia variegata*, killing 73.3% of the individuals tested. Similarly, Swaminathan et al. (64) and Ahmad et al. (65) observed that various species of coccinellid beetles died upon contact with neem oil at different concentrations.

Overall, our results indicate that karanja oil is highly toxic to parasitoids and predators when they come into direct contact with it. However, we also found that parasitism performance by *E. formosa* is not significantly affected if natural enemies do not come into direct contact with the botanical extract. This suggests that karanja oil could be potentially incorporated into IPM programs, provided that its application precedes the release of predators and parasitoids. However, it is important that bioinsecticides minimize their negative effects on non-target organisms and work synergistically with other strategies of biological control, as studies have shown that the application of botanical extracts can be antagonistic to the release of natural predators and pollinators (66). In this context, further research is needed on both the efficacy and affordability of bioinsecticides, as well as on their chemical development to ensure safe and sustainable use (67).

## 5 Conclusions

Our study focused on understanding the role of karanja oil as a toxic substance within a tri-trophic food web. The main components of this food web include tomato plants (*Solanum lycopersicum*) as primary producers, greenhouse whiteflies (*T. vaporariorum*) as first-order herbivores, and parasitoid wasps (*E. formosa*) and predatory beetles (*D. catalinae*) as natural enemies. In this food web, karanja oil disrupts the community structure through two main mechanisms: (1) by directly affecting the organisms present in the food web, and (2) by altering the ecological interactions among these organisms.

We found that karanja oil does not have a phytotoxic effect on tomato plants, but it is lethal to whitefly adults and nymphs, with toxicity depending on the contact dose. This lethal effect interrupts feeding behaviour in both adults and nymphs and reduces subsequent egg-laying, potentially increasing the marketability of greenhouse crops treated with karanja oil. Additionally, while karanja oil is lethal to parasitoids and predators upon direct contact, it does not interfere with parasitism by parasitoid wasps when they are released after the application of karanja oil. However, although we observed that the release of predator beetles after the application of karanja oil reduces the number of nymphs alive, it remains unclear whether the predation by *D. catalinae* is inhibited or enhanced. Further research is needed to clarify this interaction.

These results are relevant for integrated pest management (IPM) programs that combine the application of botanical extracts with the release of natural enemies. Our findings suggest that these activities can be used synergistically, but with a strict order of application to maximize efficacy. However, more research is needed regarding karanja oil chemical ecology for its fully implementation in such programs.

## Data Availability

The raw data supporting the conclusions of this article will be made available by the authors, without undue reservation.
