# Supplementary material for: Toxicity across food webs: effects of karanja oil on greenhouse whitefly on tomato and two commercial biocontrol agents
Source: Front Insect Sci. 2026 Feb 4;6:1711812. doi: 10.3389/finsc.2026.1711812 (PMC12914566; doi:10.3389/finsc.2026.1711812)
Supplement: Supplementary file 1 [file DataSheet1.pdf]

Supplementary material

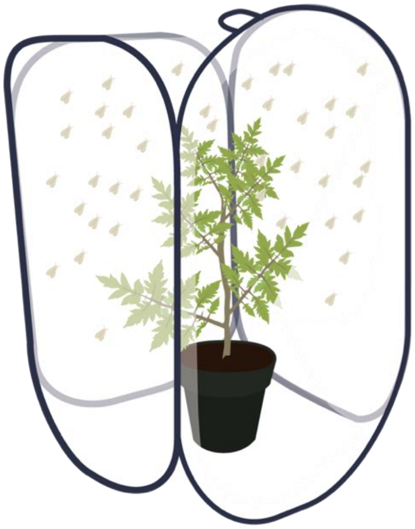

Figure S1. Trials with whole plants: 7-week-old tomato plants in mesh cages with whitefly adults *T vaporariorum*

Application of karanja oil to the plant root system

To evaluate whether a root application of karanja oil could effectively kill *T. vaporariorum* (e.g., through a systemic effect), we poured different concentrations of the solution directly onto the roots of 7-week-old tomato plants (*S. lycopersicum*). Prior to treatment application, we conducted a water pot capacity trial with 1.5-litre pots filled with substrate mix. In this trial, we added water to a pot with saturated soil until it reached the capacity level (230 ml per pot), noting that the percolation of the karanja oil solution was minimal at that capacity.

We placed the tomato plants in sealed enclosures with same-aged *T. vaporariorum* adults (n=40 for each experimental unit), using six replicates per treatment. We then poured the

karanja oil solution (1%, 2%, or 3% (v/v%)) or a water control directly into the substrate weekly for three weeks, with one application per week. To prevent treatment runoff, we installed drip irrigation lines. We counted the number of nymphs 48 hours after the last application.

Pouring karanja oil at different concentrations into the root system did not have a lethal effect on *T. vaporariorum* nymphs feeding on tomato plants (karanja: $F_{3,20}$=0.51, p=0.67; Figure 9). In summary, we did not find significant differences in the number of insect nymphs alive between treatments with different concentrations of karanja oil and control.

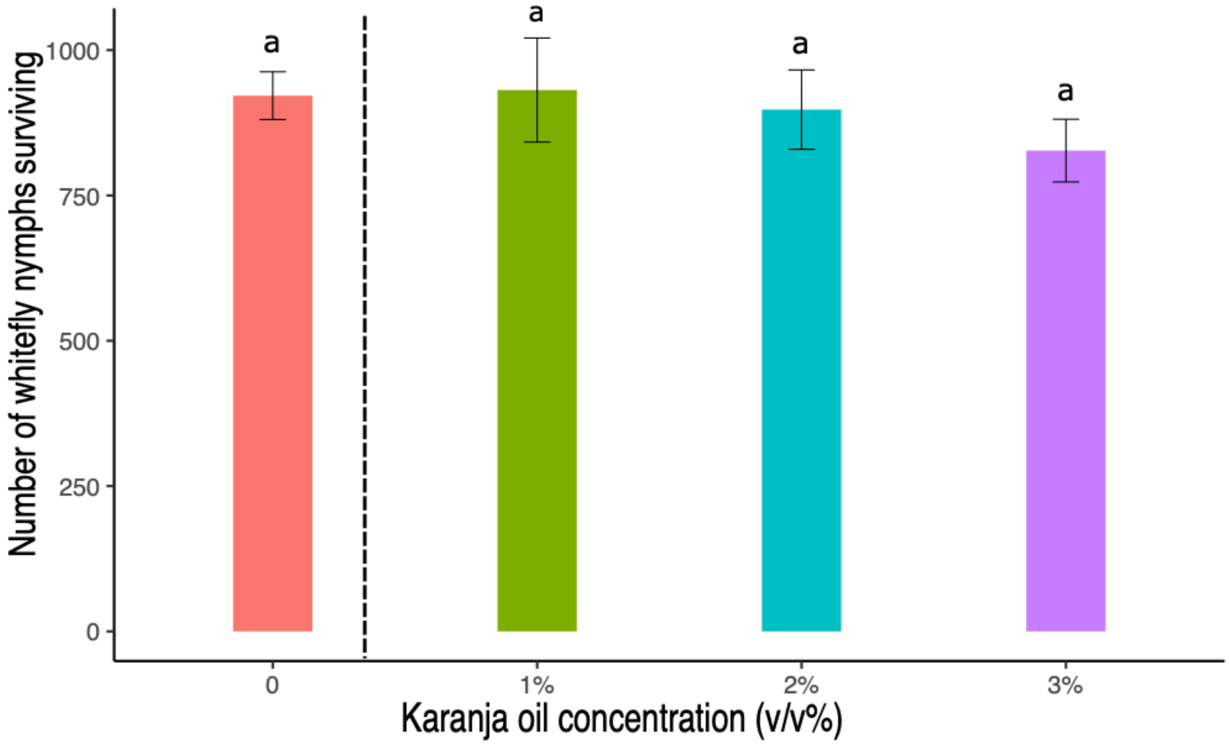

Figure S2. Number of *T. vaporariorum* nymphs surviving after 3 weeks of pouring different concentrations of karanja oil to the roots (Control, 0; 1%; 2%; 3%). Means and standard error shown. Different letters indicate significant differences.
